# A Systematic Review of Insulin Management Recommendations to Improve Glycemic Control and Reduce Hypoglycemic Events During Ramadan Fasting in Patients With Insulin-Requiring Type 2 Diabetes

**DOI:** 10.3389/fnut.2022.846600

**Published:** 2022-05-12

**Authors:** Alexander Kieu, Ashley Iles, Moien AB Khan, Linda Östlundh, Duston Boyd, MoezAlIslam Ezzat Faris

**Affiliations:** ^1^College of Medicine and Health Sciences, United Arab Emirates University, Al Ain, United Arab Emirates; ^2^Kanad Hospital, Al Ain, United Arab Emirates; ^3^School of Medicine, University of Louisville, Louisville, KY, United States; ^4^Covenant Community Care Clinic, Detroit, MI, United States; ^5^School of Medicine, Wayne State University, Detroit, MI, United States; ^6^Department of Clinical Nutrition and Dietetics, University of Sharjah, Sharjah, United Arab Emirates

**Keywords:** type 2 diabetes, Islam, insulin, hypoglycemia, hyperglycemia

## Abstract

**Background:**

Muslims with insulin-requiring type 2 diabetes are at high risk of hypo- and hyperglycemia while fasting during the month of Ramadan. Although a few reviews on diabetic management during Ramadan have been published, surveys reveal knowledge gaps remain among physicians.

**Aim:**

This systematic review qualitatively analyzes what insulin dosing recommendations are likely to reduce hypoglycemic events and improve glycemic control during the Ramadan fasting for this high-risk group.

**Methods:**

A comprehensive search in six databases and gray sources was performed from August 10, 2001, to August 10, 2021, for studies assessing which types of insulin and/or what dosing recommendations reduce hypoglycemic events and improve glycemic control during Ramadan. We excluded studies focusing mainly on oral antihyperglycemic medications, type 1 diabetes, persons with insulin pumps, and studies older than 20 years. Hypoglycemic event rates, pre-, and post-iftar blood glucose levels, overall average blood glucose, and hemoglobin A1c were analyzed, and a narrative synthesis was performed.

**Results:**

Out of 1,101 collected articles, 14 eligible studies including 2,969 participants with an average age of 54.8 years, we found that insulin dose reduction may prevent hypoglycemia without causing subsequent hyperglycemia, and rapid-acting insulin analogs may improve post-iftar and overall blood glucose without incurring hypoglycemia.

**Conclusions:**

Though initial findings are promising, more research is needed to confirm the benefits of insulin dose reduction, rapid-acting insulin analogs, and ultra-long-acting insulins.

**Systematic Review Registration:**

https://www.crd.york.ac.uk/prospero/, identifier: CRD42021268943.

## Introduction

The burden of diabetes mellitus continues to rise globally across all regions of the world (1). Approximately 463 million adults are living with diabetes worldwide, and this figure is projected to increase by 51% in the next 25 years (2). Of all the people with diabetes globally, 150 million are estimated to be Muslim (3). One of the five pillars of Islam, central to the Muslim faith, is annual fasting during the holy month of Ramadan. During this month, all healthy Muslims who have reached puberty are required to fast from dawn to sunset, which includes refraining from eating, drinking, use of oral medications, and smoking (4). Exemptions are available for certain populations, such as Muslims who are elderly, traveling, expecting, or nursing mothers and Muslims with serious medical conditions including diabetes. However, many Muslims with these conditions still voluntarily choose to observe the practice of fasting during Ramadan (5). Epidemiologic studies have shown that Muslims with diabetes fast for an average of 27–28 days in the month of Ramadan (4, 6–8). Even 43.9% of Muslims with a “high” or “very high” risk classification of diabetes (according to the American Diabetes Association, ADA) fasted for an average of 28 days during Ramadan despite medical advice (9).

Hypoglycemia during Ramadan is a major concern, particularly for those who fast for up to 20 h consecutively (4). Recurrent hypoglycemia may increase the risk of cognitive impairment and mortality (10). This concern is heightened for patients with insulin-requiring diabetes, many of whom are categorized as very high risk by the ADA risk index (9). One study showed this very high-risk cohort has a 13.8% incidence of hypoglycemia compared to 4.2% for low-risk individuals (9). The same study showed that persons with type 2 diabetes taking only insulin during Ramadan had a greater incidence (16.8%) of hypoglycemia than those treated with only oral hypoglycemic agents (5.3%) (9).

In addition to hypoglycemia, hyperglycemia during Ramadan is also a major concern. One large epidemiologic study revealed a significantly increased risk of severe hyperglycemia or ketoacidosis during Ramadan (0.05 ± 0.35) compared to before Ramadan (0.01 ± 0.05) (4). A possible explanation for this may be because the meal that breaks the fast after sunset (iftar) is typically larger than average, and it has been shown that the risk of hyperglycemia is consequently higher (6). A more recent study that used flash-glucose monitoring on insulin-treated patients during Ramadan showed an increase in time in hyperglycemia and a reduced time in the target range (11). The long-term effects of elevated uncontrolled blood glucose are well-known including increased risk of cardiovascular disease, nephropathy, neuropathy, and ophthalmopathy.

Although the Diabetes and Ramadan International Alliance (DaR) and the International Diabetes Federation (IDF) collaboratively published practice guidelines (5, 12), healthcare providers continue to have knowledge gaps pertaining to diabetes management during Ramadan. Beshyah et al. conducted a survey on 260 physicians from 27 countries—almost all Muslim majority countries. Many physicians surveyed (54.1%) admitted to having a knowledge gap in the practical management of high-risk diabetic groups, and 49.2% believed there is limited data on high-risk patients. Additionally, respondents most desired knowledge on how to best organize healthcare before, during, and after Ramadan, and if newer pharmacological agents are better than older ones if used correctly. Most physicians agreed that the two most appropriate types of articles to disseminate knowledge about Ramadan fasting are original research (73.3% of respondents) and systematic reviews (64.3%) (13).

A few reviews have analyzed both glycemic control and adverse events during Ramadan for insulin-requiring diabetes, specifically. However, to our knowledge, the original research from which these reviews and guidelines derived their recommendations have not been critically appraised. Additionally, there is no systematic review dedicated exclusively to insulin management in type 2 diabetes during Ramadan. Given the growing body of literature on Ramadan and diabetes, this systematic review seeks to answer the question: what insulin dosing recommendations are likely to reduce hypoglycemia and improve glycemic control for persons with insulin-requiring type 2 diabetes who participate in the Ramadan fast?

## Materials and Methods

The review is reported in accordance with the 2020 Preferred Reporting Items for Systematic Reviews and Meta-analyses (PRISMA) statement and informed by the Cochrane Handbook for Systematic Reviews of Interventions (14, 15). A review protocol was prospectively registered online in PROSPERO (Prospective, International Register of Systematic Reviews) under the registration number: CRD42021268943.

### Literature Search

A medical librarian specialized in systematic reviews (LÖ) conducted a comprehensive search for literature in six electronic databases: PubMed (NML), EMBASE (Elsevier), CINAHL (Ebscohost), Scopus (Elsevier), Web of Science (Clarivate), and Cochrane Library (Cochrane Collaboration). Gray literature sources were located via OAlster Gray Repository, World Health Organization Institutional Repository (WHO IRIS), ClinicalTrials.gov, BASE, and Open Gray. The search was performed in August 2021. Pre-searches in PubMed and PubMed's MeSH to identify relevant search term variations and to develop the search string was conducted by LÖ in May-July 2021 in close collaboration with subject specialists (AK and MK). The search strategy developed in PubMed was later systematically repeated in all selected information sources. A combination of the search fields title, abstract, keywords (Text Word, TOPIC, or similar), and “MeSH”/“thesaurus” (when available) was used to ensure that the best possible evidence was located. The search was conducted without any language or geographical restrictions. Because of the recent advances in diabetic management, studies older than 20 years were excluded. Search details, dates, keywords, results, and notes for all databases and gray sources are reported in Appendix A.

### Study Selection

All records identified in the database search were uploaded to the systematic review software Covidence (Veritas Health Innovation, 2021) for automatic de-duplication and prepared for blinded screening and data extraction (LÖ) (16). The results from the gray sources were de-duplicated by hand. Cabell's Predatory Report (Cabell's Scholarly Analytics, 2021) was consulted to verify the scientific status of included studies published in open access journals (17). Two independent reviewers (AK and MK) screened the titles and abstracts of unique records against the pre-set inclusion and exclusion criteria, which is summarized in Table 1.

**Table 1:** PICOS criteria applied for the systematic review.

**Parameter**	**Inclusion criteria**	**Exclusion criteria**
Population	Persons with insulin-requiring type 2 diabetes	Type 1 diabetes, Persons with an insulin pump
Intervention	All insulin types and insulin dosing strategies during Ramadan	Studies focusing mainly on oral antihyperglycemic medications
Comparison	Studies comparing insulin subtypes or insulin dosing strategies	
Outcomes	The difference in hypoglycemic event rate, pre-and post-iftar blood glucose, overall blood glucose, and HbA1c between the insulin types and/or insulin dosing strategies	Studies that do not quantify glycemic control or adverse events or report any outcomes.
Study type	Studies focusing on insulin type and/or dosing strategies in insulin-requiring type 2 diabetes during Ramadan	Reviews, epidemiological studies, editorials, case reports, conference abstracts, comments, and letters to the editors; physiologic focused articles
Time	The cut-off date limit of 2001–2021 was applied	Studies published before 2001
Language	All countries and all languages	
Setting	All settings	

Because this review aims to guide insulin management for type 2 diabetes during Ramadan, a concerted effort was made to include studies focusing on insulin and exclude studies with an emphasis on oral hypoglycemic agents. If studies were able to control for oral hypoglycemic agents and isolate the effects of insulin on hypoglycemia and glycemic control during Ramadan fasting, they were included for review. Additionally, if studies evaluated a specific insulin dosing strategy and measured its effects on blood glucose levels, they were included for review. If a study included persons with insulin-requiring type 2 diabetes during Ramadan, but the primary objective of the study was to evaluate the effects of an oral hypoglycemic agent, this study was excluded. If it was difficult to ascertain the effects of insulin specifically on diabetic management during Ramadan because an oral hypoglycemic agent was an uncontrolled variable in the study, this study was excluded.

**Figure 1:**
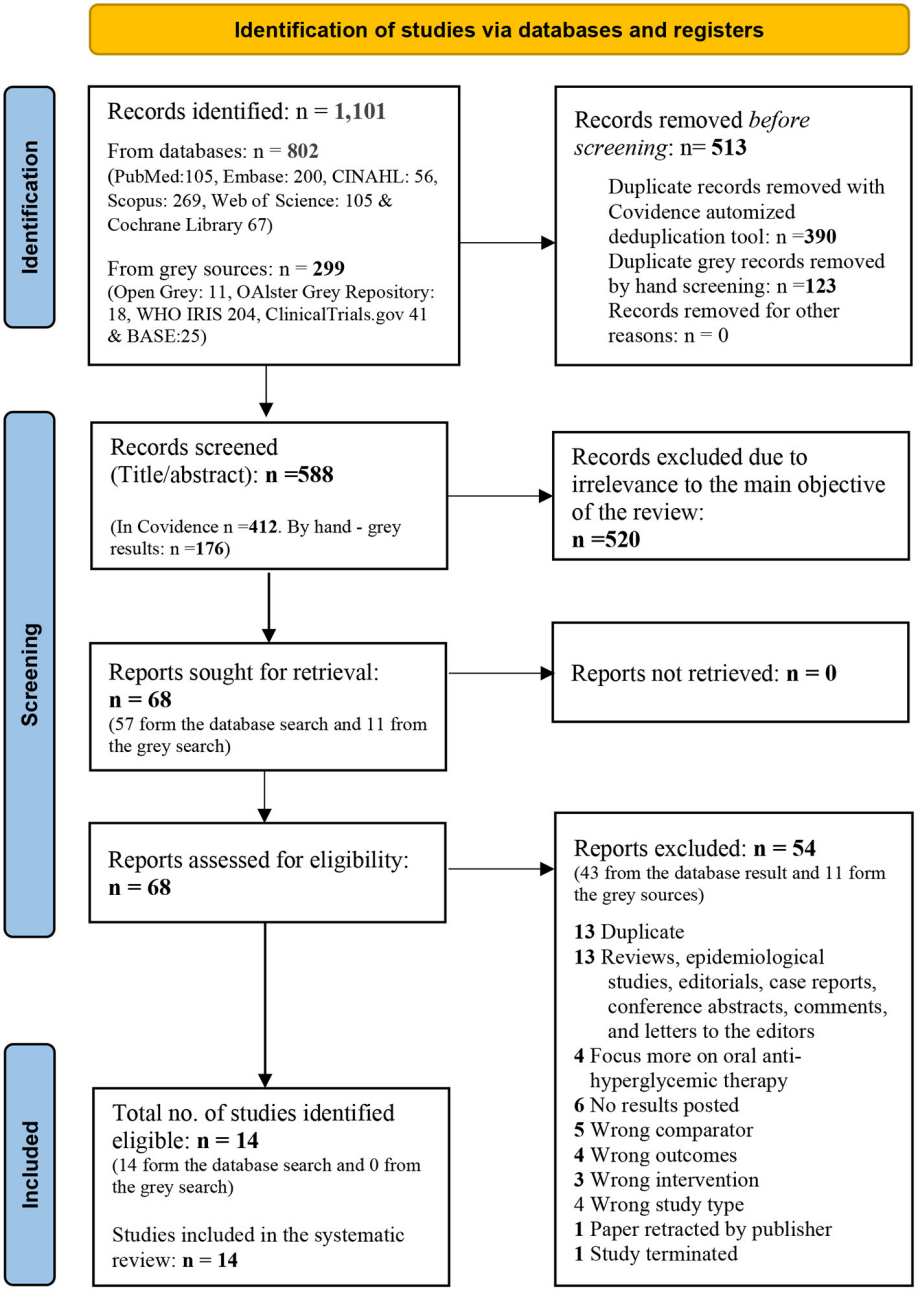
PRISMA 2020 flow diagram for new systematic reviews which included searches of databases and registers only. From: Page et al. (14). For more information, visit: http://www.prisma-statement.org/.

A third independent reviewer (AI) resolved the conflicts identified by the software. Full-text papers were sought and uploaded to Covidence for blinded screening (AK and AI) and conflict resolution (MK). A PRISMA flow diagram with details of the screening and selection process is illustrated in Figure 1.

### Data Extraction

Two independent reviewers (AK, AI) used the Covidence software to extract study characteristics and outcomes. The primary outcome assessed was the difference in hypoglycemic incidence or event rate between the insulin types and/or insulin dosing recommendations. Hypoglycemia was defined in most studies as a serum blood glucose level < 70 mg/dL (3.9 mmol/L) except in two studies it was <63 mg/dL (3.5 mmol/L), and one study defined hypoglycemia as <60 mg/dL (3.3 mmol/L). The secondary outcomes obtained reflected glycemic control which was measured by the changes in pre-and post-iftar blood glucose (mg/dL, mmol/L), overall blood glucose (mg/dL, mmol/L), and HbA1c (mmol/mol) between the insulin subtypes and/or dosing recommendations. Additionally, insulin dosing strategies were examined and compared between studies. Type 2 diabetes-associated adverse events, such as DKA or HHS was also extracted when available in addition to each study's funding sources. A third reviewer (MK) was available to resolve any conflicts if necessary.

### Quality Assessment

Two independent reviewers (AK, MK) used the Newcastle-Ottawa Scale (NOS) and the National Heart, Lung, and Blood Institute's (NHLBI) quality assessment tools to assess the quality and risk of bias of each observational cohort study and controlled intervention study, respectively (18, 19). A third reviewer (DB) resolved conflicts. The Covidence software enabled a systematic and blinded approach.

### Data Synthesis

The characteristics and outcomes of each study were summarized in a comprehensive table, allowing us to group and compare similar findings relevant to our primary and secondary outcomes. Our primary outcome, the hypoglycemic incidence or event rate, was expressed as a difference in the percentage between different insulin types and/or dosing recommendations, and the *p*-values from each study were noted. The CREED epidemiologic study reported a hypoglycemia incidence rate of 13.8% for insulin-requiring type 2 diabetes during Ramadan, so this was the benchmark value that we used in our analysis. Our secondary outcomes concerning glycemic control were measured as the mean difference in blood glucose levels between different insulin types and/or dosing recommendations while considering the *p*-value of each respective measurement. If a study found that a specific insulin subtype or dosing recommendation decreased the hypoglycemia incidence rate to <13.8% without significantly increasing post-iftar blood glucose, overall blood glucose, or severe hyperglycemia (blood glucose > 300 mg/dL, 16.7 mmol/L), this would be an acceptable finding. A narrative synthesis was done to analyze which insulin types and dosing recommendations are superior for improving glycemic control and reducing hypoglycemic events during Ramadan. Due to the high heterogeneity of study designs, interventions, and outcomes, we did not perform a meta-analysis.

## Results

Of the 1,101 records located in the literature search, 588 unique studies remained for the title and abstract screening after de-duplication. All studies were found to be in the English language. Sixty-eight papers were selected for full-text screening, of which fourteen were identified as eligible to be included in the systematic review. A PRISMA 2020 flow diagram detailing the search, de-duplication, screening, and selection process is summarized in Figure 1.

The total number of study participants was 2,969 between all fourteen studies reviewed. These studies were conducted across four continents in 25 different countries; there were six in Africa: Algeria (*n* = 1), Egypt (*n* = 3), Libya (*n* = 1), Morocco (*n* = 2), South Africa (*n* = 2), and Tunisia (*n* = 1), seventeen in Asia: Bangladesh (*n* = 1), China (*n* = 1), India (*n* = 5), Indonesia (*n* = 1), Iraq (*n* = 1), Israel (*n* = 1), Jordan (*n* = 4), Kuwait (*n* = 1), Lebanon (*n* = 3), Malaysia (*n* = 3), Oman (*n* = 1), Pakistan (*n* = 3), Qatar (*n* = 2), Saudi Arabia (*n* = 3), Singapore (*n* = 1), Turkey (*n* = 1), the United Arab Emirates (*n* = 1), one in Europe: the United Kingdom (*n* = 1), and one in North America: Canada (*n* = 1). Among all fourteen studies, the average age is 54.8 years, the average duration of diabetes is 11.1 years, 52.3% were female, and 47.7% male.

Five studies were RCTs, and the remaining nine studies were observational cohort studies. A comprehensive summary of all included studies is organized in Table 2 with RCTs in the top five rows. Six studies analyzed how insulin dosage adjustment affects glycemic control and hypoglycemia during Ramadan, three examined newer ultra-long-acting insulins, three compared insulin analogs (synthetic insulin designed to mimic the body's natural insulin release pattern) to regular human insulin, and two studied specific medical regimens for the Ramadan fast. We have grouped these studies according to the previously mentioned categories concerning hypoglycemia and glycemic control. These findings are summarized in Tables 3, 4.

**Table 2:** Comprehensive summary of included studies.

**References**	**Journal name**	**Countries**	**Study design, #participants, study duration**	**Average participant age, % female, duration of diabetes**	**Aim of study**	**Funding sources**
Hajjaji et al. (20)	Int'l Jour of Clinical Practice	Libya	RCT *n* = 40 5 weeks	53.5 years 52.5% 12.3 years	To test if changing the Iftar insulin to a 50:50 mixed analog insulin from a 30:70 human insulin improves postprandial glucose	Eli Lilly for insulin mix
Hassanein et al. (21)	Diabetes Research and Clinical Practice	Algeria, India, Lebanon, Malaysia, and South Africa	RCT *n* = 248 34 weeks	55.1 years 55.9% 12.2 years	Compare the efficacy and safety of insulin degludec/insulin aspart (IDegAsp) and biphasic insulin aspart 30 (BIAsp 30) before, during, and after Ramadan	Novo Nordisk A/S
Mattoo et al. (22)	Diabetes Research and Clinical Practice	India, Pakistan, Malaysia, Singapore, Egypt, South Africa, Morocco	RCT *n* = 151 10 weeks	53 years 54.3% 12.5 years	To compare the effects of insulin lispro Mix25 and human insulin 30/70 on the daily BG profiles, specifically the postprandial BG control	Eli Lilly
Shehadeh et al. (23)	Int'l Jour of Clinical Practice	Israel	RCT *n* = 238 2 months	59.8 years 61.4% 12.7 years	Comparing insulin detemir (Levemir) and biphasic insulin (NovoMix70) to standard care	Grant from Novo Nordisk
Zaghlol et al. (24)	Frontiers in Endocrinology	Jordan	RCT *n* = 365 on insulin 5 months	58.0 years 50.4% 8.2 years	Investigate the effect of dosage reduction of four hypoglycemic multidrug regimens on the incidences of acute glycemic complications	Not stated
Ahmedani et al. (25)	Diabetes Research and Clinical Practice	Pakistan	Cohort *n* = 54 2 months	54.7 years 51.9% 13.8 years	To observe the effect of keeping flexible glycemic targets during fasting and tighter targets during non-fasting hours in insulin-treated people with type 2 diabetes during Ramadan	Unspecified
Altemimi et al. (26)	Cureus	Iraq	Cohort *n* = 30 10 weeks	53 years 54.0% 9.3 years	To compare the degree of glycemic control, tolerability, and the existence of dysglycemic events from the use of either human premixed insulin or basal plus short-acting insulin regimens	Unspecified
Ba-Essa et al. (27)	Diabetes Research and Clinical Practice	Saudi Arabia	Cohort *n* = 360 3 months in 2015 and 2016	53.8 years 54.7% 12.5 years	To determine the safety and effect of diabetes medication on glycemic control and the risk for hypoglycemia and to find some predictors associated with increased risk for hypoglycemia during fasting.	None reported
Beano et al. (28)	Endocrinology and Metabolism	Jordan	Cohort *n* = 301 2 months	58.7 years 52.6% 7.0 years	To assess the safety of a protocol involving dose adjustments to four different anti-diabetic drug regimens in T2DM patients who chose to fast during Ramadan.	Unspecified
Elhadd et al. (29)	Diabetes Research and Clinical Practice	Qatar	Cohort *n* = 12 (on basal insulin) 20 weeks	50.8 years 15.2% 13.1 years	To assess the effect of structured education and medication dose adjustment, according to the PROFAST Protocol on the risk of hypoglycemia captured using FGM in patients on sulfonylurea or basal insulin and at least 2 other diabetes medications, before and during Ramadan.	Medical Research Council, Ministry of PH, Qatar
Hassanein et al. (30)	Diabetes Research and Clinical Practice	Kuwait, Qatar, Saudi Arabia, UAE, Jordan, Lebanon, Turkey, Egypt, India, Pakistan, Canada	Cohort *n* = 466 5 months	54.4 years 48.3% 9.1 years	To prospectively evaluate the safety and effectiveness of Gla-300 in participants with T2DM prior to, during, and after Ramadan.	Sponsored by Sanofi (Gla-300 manufacturer)
Hui et al. (31)	Int'l Jour of Clinical Practice	United Kingdom	Cohort *n* = 6 12–14 weeks	62.1 years 65.4% 9.7 years	Compare hypoglycemic events, HbA1c, and changes in body weight between Humalog Mix 50 and human Mixtard 30 twice daily	None reported
Kalra et al. (32)	Indian Jour of Endocrinology and Metabolism	India	Cohort *n* = 349 6 months	46.3 years 66.7% N/A	Document the utility and safety of insulin degludec (IDeg) and insulin degludec aspart (IDegAsp)	None
Salti et al. (33)	Diabetic Medicine	Bangladesh, China, Egypt, Kuwait, Oman, UAE, Indonesia, Lebanon, India, Jordan, Malaysia, Morocco, Saudi Arabia, Tunisia	Cohort *n* = 349 6 months	54.5 years 49.0% 11.3 years	To determine the safety and efficacy of the combination of insulin glargine and glimepiride in patients with T2DM before, during, and after Ramadan.	Sanofi-Aventis

*RR, relative risk; R, Ramadan; TDD, total daily dose (of insulin); BG, blood glucose; ERR, Estimated Relative Risk; AE, adverse events; FBG, Fasting Blood Glucose; PP, post-prandial; Exp, experimental; DKA, diabetic ketoacidosis; NKHS, non-ketotic hyperosmolar syndrome*.

**Table 3:** The effects of different insulin subtypes and dosing strategies on hypoglycemia.

**References study design**	**Intervention**	**Comparator**	**Effect on hypoglycemia**	**Dosing recommendations**
Hajjaji et al. (20) RCT	Humalog Mix 50/50 at iftar (lispro/protamine) Humalog Mix 75/25 at suhur	Human mixed insulin 30:70 (Human Mixtard 30)	3 hypoglycemic episodes in each group, considered “minor” (BG of ≤ 70 mg/dL)	Decrease short-acting dose at Suhur
Hassanein et al. (21) RCT	IDegAsp BID	BIAsp 30 BID	During R: 62% reduction in overall hypoglycemia in the IDegAsp arm (ERR 0.38, *p* = 0.007); During treatment period: IDegAsp rate of overall (ERR 0.26, *p* < 0.0001, 74% RR) and nocturnal (ERR 0.17, *p* < 0.0001, 83% RR) hypoglycemia lower. Severe hypoglycemia lower (44%, *p* = 0.5801)	Use dose adjustment and titration algorithm for dosing insulin for both efficacy and safety. IDegAsp may be safer than BIAsp 30 in Ramadan fasting (lower risk of hypoglycemia)
Mattoo et al. (22) RCT	Lispro Mix 25 × 2 weeks then human insulin 30/70 × 2 weeks	Human insulin 30/70 × 2 weeks then Lispro Mix 25 × 2 weeks	Similar rate between the two groups (0.49 ± 0.9 for lisproMix25 and 0.49 ± 0.8 for insulin 30/70; *P* = 0.725)	Insulin Lispro Mix25 had better glycemic control (overall, pre-iftar and 2 h post iftar) without increasing hypoglycemic risk compared to human insulin 30/70
Shehadeh et al. (23) RCT	60% TDD pre-R split: 40% Levemir at suhur, 60% biphasic 70 at iftar	Standard care per ADA recommendations	AE rate significantly lower in intervention group (0.04 vs. 0.07, *p* = 0.010). Hypoglycemia more common in control [6 (4.8%) vs. 24 (21.4%), p,â§0.001]	Insulin dose (intervention) was 60% of the usual, of this 40% was dosed as Levemir at sunrise and 60% as biphasic 70 before dinner
Zaghlol et al. (24) RCT	75% insulin dosage reduction	Regular dosing	Low dosage vs. regular: M+IG 3.9 vs. 20.6% [odds ratio 0.16 (0.05–0.46), *p* < 0.001], M + IG + HRI 5.2 vs. 27.6% [odds ratio 0.14 (0.05–0.39), *p* < 0.001]. No incidence of DKA or HHS	Dose decreases (75% tested in this study) did decrease hypoglycemia without increasing hyperglycemia or its adverse sequela.
Ahmedani et al. (25) Cohort	Insulin dose adjustments (100-200 mg/dl fasting, 100–180 mg/dl non-fasting	None	6 (0.6%) episodes of hypoglycemia reported; no hospitalizations for hypoglycemia.	1) switch insulin usual morning and evening doses 2) use flexible targets (100–200 mg/dl fasting, 100–180 mg/dl non-fasting hrs
Altemimi et al. (26) Cohort	Human premixed (NPH/regular) 2/3 before iftar; 1/3 before suhur	1/2 TDD of human regular short-acting before iftar, and 1/2 basal NPH	Hypoglycemic events were reported with both groups (35.7 and 43.8% of participants from premixed and basal+ short-acting, respectively), with no statistical difference.	Both regimens are effective for glycemic control and can be used safely for fasting “if the treatment is personalized on a case-by-case basis.”
Ba-Essa et al. (27) Cohort	Ramadan focused diabetes education, diet counseling	None	Insulin only (13.6%) = 46.9% hypo; Insulin + OHA (30%) = 35.2% hypo; Basal + SU (13.1%) = 29.8% hypo; Basal + Non-SU-OHA (3.3%) = 0 hypo; MDDI ± Non-SU-OHA (13.6%) = 49% hypo	Insulin increased the risk of hypoglycemia during Ramadan except when using basal + non-SU-OHA
Beano et al. (28) Cohort	Reduction by 75% in all doses (if bid, 45% iftar, 30% suhur)	None	Reduced # hypoglycemic episodes in all groups vs. preceding month, Group C (metformin+insulin): *p* = 0.008, Group D (insulin alone): *p* = 0.02	75% TDD (if bid, 45% Iftar, 30% suhur)
Elhadd et al. (29) Cohort	Reduction of basal insulin 25% and SU by 50%	Compared to sulfonylureas and itself (full dose)	No difference before or during Ramadan	Reduce insulin dose by 25% per PROFAST Ramadan protocol
Hassanein et al. (30) Cohort	Gla−300, patient education and dosing adjustment	None	No significant difference in pre, post, during R in # of episodes. No severe hypoglycemia episodes during and post-Ramadan. Daytime hypoglycemic events are more common than nocturnal.	People with T2DM using Gla-300 during R had a low risk of severe/symptomatic hypoglycemia and improved glycemic control
Hui et al. (31) Cohort	Humalog Mix 50 at iftar, Mixtard 30 suhur	Mixtard 30 bid	No statistically significant difference between rates of hypoglycemia in Mix 50 vs. Mix 30 groups.	Changing to Humalog Mix 50 for Iftar improved glycemic control without increasing hypoglycemia (maybe)
Kalra et al. (32) Cohort	IDeg or IDegAsp	None	No severe hypoglycemic episodes were reported in patients. 3 total episodes of hypoglycemia were reported in the non-fasting period and were self-treated successfully.	IDeg dose may need reduction by 25% for Ramadan. Dose reduction of 25-30% at Suhur with IDegAsp “May” switch morning dose of IDegAsp to evening meal with changing dose amt.
Salti et al. (33) Cohort	Insulin glargine and glimepiride	None	Minimal severe hypoglycemic episodes, mild hypoglycemic episodes increased from 156 pre-R and 153 post-R vs. 346 during R (*p* < 0.001, *p* = 0.0002). FBG > 6.7 mmol/L had a protective effect on hypoglycemia	This combination may be useful in some patients, provided glimepiride is given at the time of breaking the fast and insulin glargine titrated to provide FBG > 6.7 mmol, ÅÑl.

*RR, relative risk; R, Ramadan; TDD, total daily dose (of insulin); BG, blood glucose; ERR, Estimated Relative Risk; AE, adverse events; FBG, Fasting Blood Glucose; PP, post-prandial; Exp, experimental; DKA, diabetic ketoacidosis; NKHS, non-ketotic hyperosmolar syndrome*.

**Table 4:** The effects of different insulin subtypes and dosing strategies on hyperglycemia.

**References study design**	**Intervention**	**Comparator**	**Effect on hyperglycemia**	**Dosing recommendations**
Hajjaji et al. (20) RCT	Humalog Mix 50/50 at iftar (lispro/protamine) Humalog Mix 75/25 at suhur	Human mixed insulin 30:70 (Human Mixtard 30) (*n* = 20)	During R, mean pp BG in Exp group lower by 21.1 mg% (*p* < 0.001) compared to control, using ANCOVA to adjust for pre-R values of age, gender, duration of diabetes. After R, mean A1c in Exp group lower 0.4% (*p* = 0.01) vs. control. Mean fasting BG no sig difference	Insulin analog mix 50:50 is preferred for glycemic control post iftar compared to intermediate insulin (human insulin mix 30:70).
Hassanein et al. (21) RCT	IDegAsp BID (*n* = 121)	BIAsp 30 BID (*n* = 127)	Significantly lower pre-iftar (−0.54 mmol/L, *p* = 0.0247). Similar A1c reduction between 2 arms	Use dose adjustment and titration algorithm for dosing insulin for both efficacy and safety.
Mattoo et al. (22) RCT	Lispro Mix 25 x 2 weeks then human insulin 30/70 x 2 weeks	Human insulin 30/70 × 2 weeks then Lispro Mix 25 × 2 weeks	For LisproMix25 vs. Human insulin 30/70: pre iftar BG 7.19 ± 2.2 vs. 7.59 ± 2.6 mmol/l (adjusted *P* = 0.034); 2 h post dinner 10.59 ± 3.2 mmol/l vs. 11.69 ± 3.4 mmol/l (*p* = 0.0001) Evening 2 h pp excursion 3.49 ± 2.9 vs. 4.09 ± 3.2 mmol/l (adjusted *p* = 0.007)	Insulin Lispro Mix25 had better glycemic control (overall, pre-iftar and 2 h post iftar) without increasing hypoglycemic risk compared to human insulin 30/70
Shehadeh et al. (23) RCT	60% TDD pre-R: 40% Levemir at suhur, 60% biphasic 70 iftar	Standard care per ADA recommendations	No significant difference in A1c. Intervention arm non-inferior to the control. Blood-glucose > 300 mg/dL event rate mean 0.01 in intervention vs. 0.02 in control (*p* = 0.026)	Insulin dose (intervention) was 60% of the usual, of this 40% was dosed as Levemir at sunrise and 60% as biphasic 70 before dinner
Zaghlol et al. (24) RCT	75% insulin dosage reduction	Regular dosing	No statistically different difference in hyperglycemia incidence in low vs. regular dose groups	Dose decreases (75% tested in this study) did decrease hypoglycemia without increasing hyperglycemia or its adverse sequela.
Ahmedani et al. (25) Cohort	Insulin dose adjustments (100–200 mg/dl fasting, 100–180 mg/dl non-fasting	None	A1c reduction 9.21 +/- 2.05 to 8.33 +/- 1.45 (p < 0.0001); 352 (30%) hyperglycemic episodes reported, no DKA or HHS	1) switch insulin usual morning and evening doses 2) use flexible targets 100–200 mg/dl fasting, 100–180 mg/dl non-fasting hrs
Altemimi et al. (26) Cohort	Human premixed (NPH/regular) 2/3 s before iftar; 1/3 before suhur	1/2 TDD of human regular short-acting before iftar, and 1/2 basal NPH	Hyperglycemic events were reported with both groups, with no statistical difference. Both groups decreased average A1c (more in premixed), but no significant difference	Both regimens are effective for glycemic control and can be used safely for fasting “if the treatment is personalized on a case-by-case basis.”
Ba-Essa et al. (27) Cohort	Ramadan focused diabetes education, diet counseling	None	A1c reduced from 8.79 before R to 8.59 post R, (*p* = 0.022) overall (not insulin specific)	NA
Beano et al. (28) Cohort	Reduction by 75% in all doses (if bid, 45% iftar, 30% suhur)	None	No episodes of DKA nor NKHS	75% TDD (if bid, 45% Iftar, 30% suhur)
Elhadd et al. (29) Cohort	Reduction of basal insulin 25% and SU by 50%	Compared to sulphonylureas and itself	No difference in A1c or average BG before or during Ramadan	Reduce insulin dose by 25% per PROFAST Ramadan protocol
Hassanein et al. (30) Cohort	Gla−300, patient education and dosing adjustment	None	A1c fell 0.4% (±1.0%) pre to post R, Gla-300 daily dose reduced 25.6 to 24.4. Fasting plasma glucose decreased (mean change −13.5 ± 44.1)	People with T2DM using Gla-300 during R had a low risk of severe/symptomatic hypoglycemia and improved glycemic control
Hui et al. (31) Cohort	Humalog Mix 50 at iftar, Mixtard 30 suhur	Mixtard 30 bid	Mix50 mean A1c reduction 0.48% (*p* = 0.0001) Mix30 A1c increase 0.28% (*p* = 0.007). Significant after adjusting for baseline factors (*p* = 0.0004, 95% CI (0.19%, 0.62%)	Changing to Humalog Mix 50 for Iftar improved glycemic control without increasing hypoglycemia (maybe)
Kalra et al. (32) Cohort	IDeg or IDegAsp	None	5 persons who switched from either premixed or NPH resulted in a 12–25% dose reduction after 14–20 days.	IDeg dose may need reduction by 25% for R; a dose reduction of 25-30% at Suhur with IDegAsp. “May” switch morning dose of IDegAsp to evening meal
Salti et al. (33) Cohort	Insulin glargine and glimepiride	None	FBG and A1c improved for insulin naive (10.9–7.0, 8.7–7.7, *p* = 0.0002) and non-insulin naive (9.8–6.9, 8.6–7.7, *p* < 0.0001)	The combination may be useful, provided glimepiride was given at iftar; insulin glargine titrated to provide FBG > 6.7 mmol, ÅÑl.

*RR, relative risk; R, Ramadan; TDD, total daily dose (of insulin); BG, blood glucose; ERR, Estimated Relative Risk; AE, adverse events; FBG, Fasting Blood Glucose; PP, post-prandial; Exp, experimental; DKA, diabetic ketoacidosis; NKHS, non-ketotic hyperosmolar syndrome*.

### Hypoglycemia

#### Insulin Dosage Adjustment

Three studies (two RCTs) out of four demonstrated that insulin dosage reduction of between 25 and 40% TDD decreases rates of hypoglycemia for some participants (23, 24, 28). The average hypoglycemic incidence rate from these two RCTs was 22.5% [21.4% in Shehadeh et al. (23), 23% in Zaghlol et al. (24)] in the control group and 4.5% [4.8% from Shehadeh et al. (23), 4.2% in Zaghlol et al. (24)] in the intervention group. Another study compared premixed twice-daily dosing to short-acting insulin at iftar with intermediate at suhur (the meal consumed early in the morning before dawn and before fasting commences) and found no difference in hypoglycemia (26). Ahmedani et al.'s strategy of flexible glycemic targets (100–200 mg/dl during fasting hours and 100–180 mg/dl during non-fasting hours) reported only a 0.6% rate of hypoglycemia (25).

#### Long and Ultra-Long-Acting Insulins

Ultra-long-acting insulins (IDegAsp twice daily) demonstrated a 62% reduction in overall hypoglycemia compared to BIAsp 30 twice daily (*p* = 0.007) in one RCT (21) and reported no severe hypoglycemic episodes in another cohort study (32). Utilization of Gla-300 resulted in no severe hypoglycemic episodes during or post-Ramadan (30). Participants taking basal + Non-SU-OHA reported zero episodes of hypoglycemia (27). The use of nighttime Levemir with reduced total daily insulin dosing resulted in a lower adverse event rate (0.04 vs. 0.07, *p* = 0.010) and a lower hypoglycemic event rate (4.8 vs. 21.4%, *p* < 0.001) compared to usual care (23). Salti et al. found that using insulin glargine with glimepiride resulted in minimal severe hypoglycemic episodes and that keeping fasting blood glucose > 120 mg/dL (6.7 mmol/L) had a protective effect on hypoglycemia (33).

#### Insulin Analogs vs. Human Insulin

Although three studies (2 RCTs) found that rapid-acting insulin analogs can improve glycemic control, each study comparing insulin lispro/protamine to human mixed insulin found no statistically significant difference in hypoglycemic event rate between the two arms (20, 22, 31). Hajjaji et al. reported three minor hypoglycemic episodes in both the intervention (rapid-acting insulin analog) and control (human insulin) groups (20), Mattoo et al. recorded a similar number of hypoglycemic episodes (0.4 episodes per participant) for both groups (22), and Hui et al. did not record a statistically significant difference in hypoglycemic events between the group using a rapid-acting insulin analog (0.04 events per participant reduction) vs. the group using human insulin (0.15 events per participant increase) (31).

### Glycemic Control

#### Insulin Dosage Adjustment

Two RCTs and four observational cohort studies analyzed how insulin dosage adjustment affects glycemic control. Three of the studies reduced the total insulin daily dose by 25% in the experimental groups, and the result was no difference in hyperglycemia compared to regular dosing (24), no episodes of DKA or NKHS (no comparator) (28), and no difference in A1c or average blood glucose compared to regular dosing (29). Shehadeh et al. reduced TDD by 40% in their intervention group, giving 60% of the reduced dose as biphasic insulin 70 for iftar and 40% as Levemir at suhur, and their result showed no difference in A1c compared to usual care. In fact, although the insulin TDD was reduced, there was a decreased event rate of >300 mg/dL (16.7 mmol/L) blood glucose in the intervention group (*p* = 0.026) (23). Altemimi compared human premixed (NPH/regular) insulin dosed as 2/3 TDD pre-iftar and 1/3 TDD pre-suhur vs. human regular insulin and NPH dosed as $\frac{1}{2}$ TDD pre-iftar and $\frac{1}{2}$ TDD pre-suhur, respectively, and the result was no statistical difference in average A1c or hyperglycemic events (26). Ahmedani et al. allowed for flexible targets: 100–200 mg/dl during fasting hours and 100–180 mg/dl during non-fasting hours. This resulted in an A1c reduction of 0.88 (*p* < 0.0001) without any DKA or HHS (25).

#### Long and Ultra-Long-Acting Insulins

Five studies examined long and ultra-long-acting insulins and their effects on glycemic control. IDegAsp was found to significantly lower pre-iftar glucose by −8 mg/dL (−0.54 mmol/L, *p* = 0.0247) compared to BIAsp 30 (21). Similarly, Gla-300 decreased A1c (−0.4%) and fasting plasma glucose (−13.5 mg/dl) during Ramadan (30). Another small study found that 5 out of 6 participants who switched from premixed or NPH to IDeg or IDegAsp resulted in 12–25% insulin dose reduction (32). For long-acting insulin, one study used Levemir and a reduced total daily dosing strategy, which resulted in a significantly reduced > 300 mg/dL (16.7 mmol/L) event rate (23). Salti et al. found that insulin glargine with glimepiride helped improve FBG (176–124 mg/dL, 9.8–6.9 mmol/L, *p* < 0.0001) and A1c (8.6–7.7%, *p* < 0.0001) in non-insulin naïve participants (33).

#### Insulin Analogs vs. Human Insulin

Rapid-acting insulin analog mixes, such as lispro/protamine, improved glycemic control in two RCTs and one cohort study when compared to human mixed insulin. During Ramadan, lispro/protamine improved pre-iftar blood glucose 6 mg/dL (0.4 mmol/L, *p* = 0.034) (22), 2-h post iftar blood glucose 20 mg/dL (1.1 mmol/L, *p* = 0.0001) (20), mean postprandial blood glucose 21.1 mg/dL (1.2 mmol/L, *p* < 0.001) (20), mean A1c 0.4% (*p* = 0.01) (31), and overall blood glucose 9 mg/dL (0.6 mmol/L, *p* = 0.004) compared to human insulin 30/70 (22). Another cohort study demonstrated improved A1c by 0.48% (*p* = 0.0001) when using lispro/protamine mix 50 at iftar as opposed to an increase in A1c by 0.28% (*p* = 0.007) in the comparator group using human insulin mix 30 at iftar (31).

### Risk of Bias and Quality Assessment

The NOS was used for the risk of bias and quality assessment for the nine included observational cohort studies. Five out of nine cohort studies were rated as *Good* according to the NOS scale, and the remaining four cohort studies were rated as *Poor*. Most cohort studies rated as *Poor* were rated as such because the analytic design of the study did not control for confounders. The NHLBI quality assessment was used for the five RCTs with two studies rated as *Fair*, and three studies rated as *Good*. Tables 5, 6 summarize our ratings for the nine observational cohort studies and the five RCTs, respectively.

**Table 5:** Newcastle-Ottawa Scale for the risk of bias and quality assessment of cohort studies.

**No**.	**References**	**Selection**	**Comparability**	**Outcome/exposure**	**Total (out of 9)**	**Quality**
1	Ahmedani et al. (25)	4	0	2	6	Poor
2	Altemimi et al. (26)	4	1	3	8	Good
3	Ba-Essa et al. (27)	4	0	3	7	Poor
4	Beano et al. (28)	3	1	2	6	Good
5	Elhadd et al. (29)	3	2	2	7	Good
6	Hassanein et al. (30)	4	0	2	6	Poor
7	Hui et al. (31)	3	2	3	8	Good
8	Kalra et al. (32)	3	0	1	4	Poor

**Table 6:** NHLBI risk of bias and quality assessment for controlled intervention studies.

**Question**	**Hajjaji et al**.	**Hassanein et al**.	**Mattoo et al**.	**Shehadeh et al**.	**Zaghlol et al**.
1	Yes	Yes	Yes	Yes	Yes
2	No	Yes	Yes	No	Yes
3	No	Yes	NR	No	Yes
4	No	No	No	No	No
5	No	No	No	No	Yes
6	Yes	Yes	Yes	No	Yes
7	Yes	Yes	Yes	Yes	Yes
8	Yes	Yes	Yes	Yes	Yes
9	Yes	Yes	Yes	Yes	Yes
10	NR	Yes	Yes	Yes	NR
11	Yes	Yes	Yes	Yes	Yes
12	No	NR	NR	Yes	Yes
13	Yes	Yes	Yes	Yes	Yes
14	Yes	Yes	Yes	No	NR
Total	8	11	10	8	11
**Quality Rating**	**Fair**	**Good**	**Good**	**Fair**	**Good**

*NR, Not reported*.

*Questions for the NHLBI risk of bias and quality assessment:*

*1. Was the study described as randomized, a randomized trial, a randomized clinical trial, or an RCT?*

*2. Was the method of randomization adequate (i.e., use of randomly generated assignment)?*

*3. Was the treatment allocation concealed (so that assignments could not be predicted)?*

*4. Were study participants and providers blinded to treatment group assignment?*

*5. Were the people assessing the outcomes blinded to the participants' group assignments?*

*6. Were the groups similar at baseline on important characteristics that could affect outcomes (e.g., demographics, risk factors, co-morbid conditions)?*

*7. Was the overall drop-out rate from the study at the endpoint 20% or lower than the number allocated to treatment?*

*8. Was the differential drop-out rate (between treatment groups) at the endpoint 15 percentage points or lower?*

*9. Was there high adherence to the intervention protocols for each treatment group?*

*10. Were other interventions avoided or similar in the groups (e.g., similar background treatments)?*

*11. Were outcomes assessed using valid and reliable measures, implemented consistently across all study participants?*

*12. Did the authors report that the sample size was sufficiently large to be able to detect a difference in the main?*

*the outcome between groups with at least 80% power?*

*13. Were outcomes reported or subgroups analyzed prespecified (i.e., identified before analyses were conducted)?*

*14. Were all randomized participants analyzed in the group to which they were originally assigned, i.e., did they use an intention-to-treat analysis?*

## Discussion

Despite the risks involved, up to 43.9% of Muslims with high-risk diabetes choose to fast during Ramadan (9, 10). Although large epidemiologic studies have demonstrated increased hypo- and hyperglycemia during the fasting month (6–9), most physicians acknowledge inexperience with managing diabetes during Ramadan (13). It is incumbent upon all physicians to identify safe and effective insulin dosing recommendations during Ramadan for Muslims, a cohort claiming almost one-third of all persons with diabetes worldwide (3). In this review, we found that insulin dosing adjustment and long and ultra-long acting insulins can reduce hypoglycemic events, and rapid-acting insulin analogs can improve post-iftar and overall blood glucose during Ramadan.

### Insulin Dosage Adjustment

Reducing the pre-Ramadan TDD of insulin by 25–40% during the fasting month appears to effectively decrease the rate of hypoglycemia (23, 24, 28) from a combined average incidence rate of 22.5–4.5% (23, 24, 28). By comparison, the CREED epidemiologic study recorded a hypoglycemia incidence rate of 13.8% in a similar demographic during Ramadan (7). Theoretically, lowering the insulin dose may consequently increase rates of hyperglycemia, however, studies have shown that decreasing the pre-Ramadan TDD of insulin does not subsequently increase the rate of hyperglycemia (23, 24), DKA (28), or A1c (23, 29). In fact, the large epidemiologic EPIDIAR study reported a severe hyperglycemic event rate of 4% during Ramadan compared to a 3% event rate in the intervention group in Shehadeh et al.'s study (23). This finding can be explained by the strategy of administering the long-acting insulin at suhur prior to the daytime fast, thus preventing hypoglycemia, and giving a higher insulin dose with the larger meal (iftar), which helps mitigate a large glucose load.

Previous reviews have similar recommendations: the IDF/DAR guidelines (5), the South Asian Health Foundation (UK) guidelines (34), Ibrahim et al. applied principles of the ADA/EASD consensus guidelines in 2020 (35), and Sadikot et al. (36) all recommend reducing the basal dose by 15–30% and the suhur dose by 25–50%. After a comprehensive systematic review and critical appraisal of the original research, we have also found that reducing the TDD of insulin is a dosing recommendation that may help reduce hypoglycemic events during Ramadan. However, it must be acknowledged that we found and reviewed only four studies relating to insulin dose adjustment, limiting our confidence in this conclusion.

Novel insulin dosing strategies may lead to improved outcomes for persons with diabetes during Ramadan. The IDF/DAR guidelines in 2021 relied on standard glycemic targets through the suhur, pre-iftar, and post-iftar periods (90–130 mg/dL) (5). However, the study done by Ahmedani and colleagues opens the door for more research regarding the possible superiority of flexible glycemic targets up to 200 mg/dL during fasting hours and tightened glycemic targets to <180 mg/dL during non-fasting hours (25). Their result of only a 0.6% hypoglycemia incidence rate appears very promising compared to the 9.2% incidence rate in a similar demographic in the CREED study (7). Future studies will need to examine the long-term impact of annually recurring permissive hyperglycemia as the study by Ahmedani et al. was only 2 months in duration.

### Long and Ultra-Long-Acting Insulins

Long and ultra-long-acting insulins, when given at sunrise, may reduce the risk of hypoglycemia during Ramadan (27, 30, 33), particularly when compared to intermediate-acting insulins (21, 23, 30). Long-acting insulins have a more attenuated peak and can accommodate fasting hours lasting as long as 20 h (37), thus resulting in fewer episodes of hypoglycemia. However, regarding hyperglycemia, each study recorded a beneficial, but different, outcome [lower pre-iftar glucose (21), lower A1c (30), insulin dose reduction (32), and reduced hyperglycemia > 300 mg/dL, 16.7 mmol/L (23)]. Because we cannot compare these outcomes to each other, we are unable to conclude long and ultra-long-acting insulins and their effects on hyperglycemia. Additionally, three of the six studies examining long and ultra-long-acting insulins had insufficient quality per the NOS, further limiting our confidence (27, 30, 32). More high-quality RCTs need to confirm whether long and ultra-long-acting insulins reduce hypoglycemic events and improve glycemic control during Ramadan.

### Rapid-Acting Insulin Analogs

Between two RCTs and one cohort study, rapid-acting insulin analogs significantly improved post-iftar blood glucose (20, 31) and overall blood glucose compared to regular human insulin without the risk of increased hypoglycemia (20, 22, 31). In 2015, Lessan et al. demonstrated that persons with insulin-treated diabetes (with/without oral antidiabetic drugs) recorded a significant difference in the mean amplitude of glycemic excursion during Ramadan (176 mg/dL, 9.8 mmol/L) compared to persons without diabetes (44 mg/dL, 2.4 mmol/L) (38). Other groups on only oral antidiabetic drugs did not show a significant difference (38). Physiologically, rapid-acting insulin analogs can better counteract these post-iftar excursions than regular-acting ones. Our findings are consistent with the previous reviews (3, 34–36), but because our review included only three relevant studies, two of which funded by industry, further research is necessary to confirm that rapid-acting insulin analogs improve glycemic control without increasing hypoglycemic events during Ramadan.

The strength of this review is that it is, to our knowledge, the only systematic review exclusively analyzing insulin subtypes and dosing strategies for insulin-treated type 2 diabetes during Ramadan. A comprehensive search of relevant literature was conducted, including gray literature, and a critical appraisal of the original research was performed. We reviewed recent literature which includes an investigation of second-generation basal insulin and the use of flexible glycemic targets. Acknowledged limitations include heterogeneity of study designs and study outcomes which precluded rigorous meta-analysis. We were able to group similar interventions and comparators between studies but given the variety of insulin types and dosing recommendations, our conclusions are limited. Additionally, six of the fourteen included studies were funded by industry which may introduce bias.

This review did not examine how combining insulin with other non-insulin antidiabetic medications affects hypoglycemic events and glycemic control. Abdelrahim et al. performed a large comprehensive review in 2021 and found that certain oral hypoglycemic agents combined with insulin are preferable in preventing hypoglycemic events and improving glycemic control during Ramadan, namely non-sulfonylureas such as incretin mimetics (39). In this review, we have shed light on which types of insulin and dosing strategies are beneficial during Ramadan. However, practically, many regimens include both insulin and oral hypoglycemic agents, so further research on the superiority of insulin subtypes and dosing strategies in combination with oral hypoglycemic agents during Ramadan would help close the knowledge gap which physicians have expressed (14).

## Conclusions

The research and body of literature pertaining to insulin-requiring diabetes and Ramadan remain sparse. Many reviews and guidelines have been published, but the original research had not been critically appraised, which we have done here. Insulin dose reduction may prevent hypoglycemic events, and rapid-acting insulin analogs may improve glycemic control without incurring subsequent hypoglycemia during Ramadan. However, more randomized controlled trials need to be performed before conclusions can be made. Though initial findings are promising, more research is needed to confirm the benefits of ultra-long-acting insulins as well as the use of flexible glycemic targets. While certain types of insulin and particular dosing strategies demonstrate some advantages, these recommendations should be tailored to the context of each person with diabetes to make the appropriate regimen adjustments in preparation for intensive fasting practices during the month of Ramadan.

## Data Availability Statement

The original contributions presented in the study are included in the article/Supplementary Material, further inquiries can be directed to the corresponding author/s.

## Author Contributions

AK conceived of the presented idea. AK, AI, MK, and LÖ designed the review. LÖ conducted the literature search and prepared the articles for screening. AK and MK conducted the title/abstract screening while AI resolved conflicts. AK and AI did the full-text screen with MK resolving conflicts. AK and AI extracted data. AK and MK performed the quality assessment with DB resolving conflicts. AK, AI, and LÖ wrote the manuscript. DB and MF designed tables. All authors edited the manuscript, discussed the results, analyzed the data, and contributed to the final manuscript. All authors contributed to the article and approved the submitted version.

## Conflict of Interest

The authors declare that the research was conducted in the absence of any commercial or financial relationships that could be construed as a potential conflict of interest.

## Publisher's Note

All claims expressed in this article are solely those of the authors and do not necessarily represent those of their affiliated organizations, or those of the publisher, the editors and the reviewers. Any product that may be evaluated in this article, or claim that may be made by its manufacturer, is not guaranteed or endorsed by the publisher.
