# Supplementary material for: A Systematic Review of Insulin Management Recommendations to Improve Glycemic Control and Reduce Hypoglycemic Events During Ramadan Fasting in Patients With Insulin-Requiring Type 2 Diabetes
Source: Front Nutr. 2022 May 12;9:846600. doi: 10.3389/fnut.2022.846600 (PMC9135391; doi:10.3389/fnut.2022.846600)
Supplement: Supplementary file 1 [file Data_Sheet_1.docx]

**Appendix A**

**Literature searches**

| **Source and search date** | **Search string** | **Results** | **Notes** |
| --- | --- | --- | --- |
| **PubMed**  (NLM)  **Coverage:**  From inception to search date  **Search date:**  2021-08-04 | (("Diabetes Mellitus, Type 2"[Mesh] OR "diabetes mellitus Type 2"[Text Word] OR "diabetes mellitus type II” [Text Word] OR "diabetes type 2"[Text Word] OR "type 2 diabetes"[Text Word] OR "diabetes type II"[Text Word] OR "type II diabetes"[Text Word] OR “DMT2”[Text Word] OR “T2DM”[Text Word]) AND ("insulins"[Mesh] OR insulin*[Text Word] OR novolin[Text Word] OR iletin[Text Word] OR glargine[Text Word] OR detemir[Text Word] OR degludec[Text Word] OR lispro[Text Word] OR aspart[Text Word] OR glulisine[Text Word] OR “B28-asp” [Text Word] OR “B28 asp” [Text Word] OR “B28asp” [Text Word] OR “novolog” [Text Word] OR “novorapid” [Text Word] OR apidra[Text Word] OR “NPH” [Text Word] OR humilin[Text Word] OR humalog[Text Word] OR semilente[Text Word] OR Detemir[Text Word] OR “NN304”[Text Word] OR “NN-304”[Text Word] OR “NN 304”[Text Word] OR tetradecanoyl[Text Word] OR “12C-Lys(B29)-DB30I”[Text Word] OR levemir[Text Word] OR lantus[Text Word] OR solostar[Text Word] OR basaglar[Text Word] OR “HOE 901”[Text Word] OR “901, HOE”[Text Word] OR “HOE-901”[Text Word] OR “HOE901”[Text Word] OR isophane[Text Word] OR protamine[Text Word] OR protophane[Text Word] OR protophan[Text Word] OR humulin[Text Word] OR insulatard[Text Word] OR monotard[Text Word] OR lente[Text Word] OR ultralente[Text Word] OR proinsulin[Text Word])) AND (ramadan[Text Word] OR ramadhan[Text Word] OR ramadaan[Text Word] OR ramazan[Text Word] OR iftar[Text Word] OR "islamic fast*"[Text Word] OR "muslim fast*"[Text Word] OR "religious fast*"[Text Word] OR suhoor[Text Word] OR sahūr[Text Word] OR suhūr[Text Word] OR sahrī[Text Word] OR sehri[Text Word] OR "breaking fast" [Text Word]) | **105** | All search terms are searched in the fields: “Text Word” and in MeSH (when available).  No filters or limitations applied. |
| **Embase**  (Elsevier)  **Source**s: All sources included  **Coverage:**  From inception to search date  **Search date:**  2021-08-04 | (('diabetes mellitus type 2':ti,ab,kw OR 'diabetes mellitus type ii':ti,ab,kw OR 'diabetes type 2':ti,ab,kw OR 'type 2 diabetes':ti,ab,kw OR 'diabetes type ii':ti,ab,kw OR 'type ii diabetes':ti,ab,kw OR dmt2:ti,ab,kw OR t2dm:ti,ab,kw OR 'non insulin dependent diabetes mellitus'/exp) AND (insulin*:ti,ab,kw OR novolin:ti,ab,kw OR iletin:ti,ab,kw OR glargine:ti,ab,kw OR degludec:ti,ab,kw OR lispro:ti,ab,kw OR aspart:ti,ab,kw OR glulisine:ti,ab,kw OR 'b28-asp':ti,ab,kw OR 'b28 asp':ti,ab,kw OR b28asp:ti,ab,kw OR 'novolog':ti,ab,kw OR 'novorapid':ti,ab,kw OR apidra:ti,ab,kw OR nph:ti,ab,kw OR humilin:ti,ab,kw OR humalog:ti,ab,kw OR semilente:ti,ab,kw OR detemir:ti,ab,kw OR 'nn304':ti,ab,kw OR 'nn-304':ti,ab,kw OR 'nn 304':ti,ab,kw OR tetradecanoyl:ti,ab,kw OR '12c lys b29:-db30i':ti,ab,kw OR levemir:ti,ab,kw OR lantus:ti,ab,kw OR solostar:ti,ab,kw OR basaglar:ti,ab,kw OR 'hoe 901':ti,ab,kw OR '901, hoe':ti,ab,kw OR 'hoe-901':ti,ab,kw OR 'hoe901':ti,ab,kw OR isophane:ti,ab,kw OR protamine:ti,ab,kw OR protophane:ti,ab,kw OR protophan:ti,ab,kw OR humulin:ti,ab,kw OR insulatard:ti,ab,kw OR monotard:ti,ab,kw OR lente:ti,ab,kw OR ultralente:ti,ab,kw OR proinsulin:ti,ab,kw OR 'insulin derivative'/exp) AND (ramadan:ti,ab,kw OR ramadhan:ti,ab,kw OR ramadaan:ti,ab,kw OR ramazan:ti,ab,kw OR iftar:ti,ab,kw OR 'islamic fast*':ti,ab,kw OR 'muslim fast*':ti,ab,kw OR 'religious fast*':ti,ab,kw OR suhoor:ti,ab,kw OR sahūr:ti,ab,kw OR suhūr:ti,ab,kw OR sahrī:ti,ab,kw OR sehri:ti,ab,kw OR 'breaking fast':ti,ab,kw OR 'ramadan'/exp OR 'religious fasting'/exp OR 'ramadan fasting'/exp)) | **200** | All search terms are searched in the fields: “title” and “abstract” (here marked with “:ab,ti”) and in the “Emtree” (here marked with “/de”) when available.  No filters or limitations applied.  Thesaurus term variation compared to PubMed’s MeSH applied as per referrals in Embase’s Emtree. |
| **Scopus**  (Elsevier)  **Coverage:**  From inception to search date  **Search date:**  2021-08-04 | (TITLE-ABS-KEY ("diabetes mellitus type 2" OR "diabetes mellitus type II" OR "diabetes type 2" OR*“*type 2 diabetes" OR "diabetes type II" OR "type II diabetes" OR "DMT2" OR "T2DM")AND (TITLE-ABS-KEY (insulin* OR novolin ORiletin OR glargine OR detemir OR degludec ORlispro OR aspart OR glulisine OR "B28 Asp" OR "B28Asp" OR "B28asp" OR "NovoLog" OR "NovoRapid" OR apidra OR "NPH" OR humilin ORhumalog OR semilente OR detemir OR "NN304" OR "NN-304" OR "NN 304" OR tetradecanoyl OR "12C-Lys(B29)-DB30I" OR levemir OR lantus OR solostar OR basaglar OR "HOE 901" OR "901, HOE" OR "HOE 901" OR "HOE901" OR isophane OR "protamine" OR protophane OR protophan OR humulin OR insulatard OR monotard OR lente OR ultralente OR proinsulin) AND (TITLE-ABS-KEY (ramadan OR ramadhan OR ramadaan OR ramazan OR iftar OR "Islamic fast*" OR "muslim fast*" OR "religious fast*" OR*s*uhoor OR sahūrOR suhūr OR sahrī OR sehri OR "breaking fast")) | **269** | All search terms are searched in the fields: “title”, “abstract” and “keywords (here marked with “TITLE-ABS-KEY”)  No thesaurus available.  No filters or limitations applied. |
| **Cochrane Library**  (Cochrane Collaboration)  **Coverage:**  From inception to search date  **Search date:**  2021-08-04 | ((MeSH descriptor: [Insulins] explode all trees) OR (insulin* OR novolin OR iletin OR glargine OR detemir OR degludec OR lispro OR aspart OR glulisine OR “B28-asp” OR “B28 asp” OR “B28asp” OR “novolog” OR “novorapid” OR apidra OR “NPH” OR humilin OR humalog OR semilente OR detemir OR “NN304” OR “NN-304” OR “NN 304” OR tetradecanoyl OR “12C-Lys(B29)-DB30I” OR levemir OR lantus OR solostar OR basaglar OR “HOE 901” OR “901, HOE” OR “HOE-901” OR “HOE901” OR isophane OR “protamine” OR protophane OR protophan OR humulin OR insulatard OR monotard OR kente OR ultralente OR proinsulin:ti,ab,kw) AND (MeSH descriptor: [Diabetes Mellitus, Type 2] explode all trees) OR (“diabetes mellitus type 2" OR "diabetes mellitus type II” OR "diabetes type 2" OR "type 2 diabetes" OR "diabetes type II" OR "type II diabetes" OR DMT2 OR T2DM):ti,ab,kw) AND (ramadan OR ramadhan OR ramadaan OR ramazan OR iftar OR "islamic fast*" OR "muslim fast*" OR "religious fast*" OR suhoor OR sahūr OR suhūr OR sahrī OR sehri OR "breaking fast":ti,ab,kw)) | **67** | All search terms are searched in the fields: “title”,“abstract” and “keywords”(here marked with :ti,ab,kw) and in MeSH (when available).  A filters for “Trials only” applied.  No MeSH term variations compared to PubMed’s MeSH |
| **Web of Science-** Core Collection  (Clarivate)  **Coverage:**  From inception to search date  **Search date:**  2021-08-04 | ((TS=("diabetes mellitus type 2" OR "diabetes mellitus type II” OR "diabetes type 2" OR "type 2 diabetes" OR "diabetes type II" OR "type II diabetes" OR DMT2 OR T2DM) AND (TS=(insulin* OR novolin OR iletin OR glargine OR detemir OR degludec OR lispro OR aspart OR glulisine OR “B28-asp” OR “B28 asp” OR “B28asp” OR “novolog” OR “novorapid” OR apidra OR “NPH” OR humilin OR humalog OR semilente OR detemir OR “NN304” OR “NN-304” OR “NN 304” OR tetradecanoyl OR “12C-Lys(B29)-DB30I” OR levemir OR lantus OR solostar OR basaglar OR “HOE 901” OR “901, HOE” OR “HOE-901” OR “HOE901” OR isophane OR “protamine” OR protophane OR protophan OR humulin OR insulatard OR monotard OR kente OR ultralente OR proinsulin) AND (TS=(ramadan OR ramadhan OR ramadaan OR ramazan OR iftar OR "islamic fast*" OR "muslim fast*" OR "religious fast*" OR suhoor OR sahūr OR suhūr OR sahrī OR sehri OR "breaking fast")) | **105** | All search terms are searched in the field: “Topic” (including title, abstract and author supplied keywords, here marked with “TS”).  No thesaurus available  No filters or limitations applied. |
| **CINAHL Complete**  (EBSCO)  **Coverage:**  From inception to search date  **Search date:**  2021-08-04 | ((TI (ramadan OR ramadhan OR ramadaan OR ramazan OR iftar OR "islamic fast*" OR "muslim fast*" OR "religious fast*" OR suhoor OR sahūr OR suhūr OR sahrī OR sehri OR "breaking fast") OR AB (ramadan OR ramadhan OR ramadaan OR ramazan OR iftar OR "islamic fast*" OR "muslim fast*" OR "religious fast*" OR suhoor OR sahūr OR suhūr OR sahrī OR sehri OR "breaking fast" ) AND ("MH "Insulins+") OR TI (insulin* OR novolin OR iletin OR glargine OR detemir OR degludec OR lispro OR aspart OR glulisine OR “B28-asp” OR “B28 asp” OR “B28asp” OR “novolog” OR “novorapid” OR apidra OR “NPH” OR humilin OR humalog OR semilente OR detemir OR “NN304” OR “NN-304” OR “NN 304” OR tetradecanoyl OR “12C-Lys(B29)-DB30I” OR levemir OR lantus OR solostar OR basaglar OR “HOE 901” OR “901, HOE” OR “HOE-901” OR “HOE901” OR isophane OR “protamine” OR protophane OR protophan OR humulin OR insulatard OR monotard OR kente OR ultralente OR proinsulin) OR AB (insulin* OR novolin OR iletin OR glargine OR detemir OR degludec OR lispro OR aspart OR glulisine OR “B28-asp” OR “B28 asp” OR “B28asp” OR “novolog” OR “novorapid” OR apidra OR “NPH” OR humilin OR humalog OR semilente OR detemir OR “NN304” OR “NN-304” OR “NN 304” OR tetradecanoyl OR “12C-Lys(B29)-DB30I” OR levemir OR lantus OR solostar OR basaglar OR “HOE 901” OR “901, HOE” OR “HOE-901” OR “HOE901” OR isophane OR “protamine” OR protophane OR protophan OR humulin OR insulatard OR monotard OR kente OR ultralente OR proinsulin) AND (MH "Diabetes Mellitus, Type 2") OR (diabetes mellitus type 2" OR "diabetes mellitus type II” OR "diabetes type 2" OR "type 2 diabetes" OR "diabetes type II" OR "type II diabetes" OR DMT2 OR T2DM)) | **56** | All search terms are searched in the fields: “title”, “abstract” (here marked with “TI and “AB”) and in “CINAHL Subject Headings” (here marked with “MH”) when available.  No filters or limitations applied.  Subject Headings variation compared to PubMed’s MeSH applied as per referrals in CINAHL Subject Headings |
| **Total number of records identified:** | | | **802** |
| **Total number of unique records identified after automatic de-duplication in Covidence:** | | | **412** |

**Grey sources**

| **Source and search date** | **Search strategy** | **Results** | **Notes** |
| --- | --- | --- | --- |
| **Source:**  Clinical Trials.gov  (US National Library of Medicine)  **Search date:** 2021-08-10 | ((ramadan OR ramadhan OR ramadaan OR ramazan OR iftar OR "Islamic fast*" OR "muslim fast*" OR "religious fast*" OR suhoor OR sahūr OR suhūr OR sahrī OR sehri OR "breaking fast") AND (type 2 diabetes))  ((ramadan OR ramadhan OR ramadaan OR ramazan OR iftar OR "Islamic fast*" OR "muslim fast*" OR "religious fast*" OR suhoor OR sahūr OR suhūr OR sahrī OR sehri OR "breaking fast") AND (insulin*)) | **27**  **14** | Only limited search functions available |
| **Source:**  WHO IRIS- Institutional Repository for information sharing. (World Health Organization)  **Search date:** 2021-08-10 | “type 2” AND ramadan  insulin* AND ramadan | **94**  **110** | Only limited search functions available |
| **Source:**  Open Grey  (INIST-CNRS)  **Search date:** 2021-08-10 | insulin* AND ramadan  “type 2” AND ramadan | **3**  **8** | Only limited search functions available |
| **Source:**  OAlster Grey Repository  (OCLC)  **Search date:** 2021-08-10 | ti:insulin* AND ramadan  ti:“type 2” AND ramada | **3**  **15** | Search in the search filed “title” (here marked with “ti”) |
| **Source:**  BASE  (Bielefeld University Library)  **Search date:** 2021-08-10 | (insulin AND ramadan) AND ("type 2 diabetes") | **25** | Search in the search filed “entire document”  Published articles excluded |
| **Total no. of records identified:** | | **299** | |
| **Total no. of unique records identified after de-duplication by hand:** | | **176** | |
